# The global cardiovascular magnetic resonance registry (GCMR) of the society for cardiovascular magnetic resonance (SCMR): its goals, rationale, data infrastructure, and current developments

**DOI:** 10.1186/s12968-016-0321-7

**Published:** 2017-01-20

**Authors:** The Global Cardiovascular Magnetic Resonance Registry (GCMR) Investigators, Raymond Y. Kwong, Steffen E. Petersen, Jeanette Schulz-Menger, Andrew E. Arai, Scott E. Bingham, Yucheng Chen, Yuna L. Choi, Ricardo C. Cury, Vanessa M. Ferreira, Scott D. Flamm, Kevin Steel, W. Patricia Bandettini, Edward T. Martin, Leelakrishna Nallamshetty, Stefan Neubauer, Subha V. Raman, Erik B. Schelbert, Uma S. Valeti, Jie Jane Cao, Nathaniel Reichek, Alistair A. Young, Lyuba Fexon, Misha Pivovarov, Victor A. Ferrari, Orlando P. Simonetti

**Affiliations:** 1 Department of Medicine, Brigham and Women’s Hospital, Cardiovascular Division, Boston, USA; 2 Harvard Medical School, 75 Francis Street, Boston, MA 02115 USA; 3 William Harvey Research Institute, London, UK; 4 Charite Universitatsmedizin, Berlin, Germany; 5 National Heart Lung and Blood Institute, Maryland, USA; 6 Revere Health, Provo, USA; 7 West China Hospital, Chengdu, China; 8 Miami Cardiac and Vascular Institute, Miami, USA; 9 University of Oxford, Oxford, UK; 10 Cleveland Clinic, Cleveland, USA; 11 San Antonio Military Medical Center, San Antonio, USA; 12 Oklahoma Heart Institute, Oklahoma, USA; 13 University of South Florida, Miami, USA; 14 Ohio State University Wexner Medical Center, Cleveland, USA; 15 University of Pittsburgh, Pittsburgh, USA; 16 University of Minnesota, Minnesota, USA; 17 St. Francis Hospital, New York, USA; 18 University of Auckland, Auckland, New Zealand; 19 Massachusetts General Hospital, Boston, USA; 20 University of Pennsylvania, Philadelphia, USA; 21 Ohio State University, Columbus, USA

**Keywords:** Registry, Cardiovascular magnetic resonance, Imaging, Patient management, Therapeutic implications

## Abstract

**Background:**

With multifaceted imaging capabilities, cardiovascular magnetic resonance (CMR) is playing a progressively increasing role in the management of various cardiac conditions. A global registry that harmonizes data from international centers, with participation policies that aim to be open and inclusive of all CMR programs, can support future evidence-based growth in CMR.

**Methods:**

The Global CMR Registry (GCMR) was established in 2013 under the auspices of the Society for Cardiovascular Magnetic Resonance (SCMR). The GCMR team has developed a web-based data infrastructure, data use policy and participation agreement, data-harmonizing methods, and site-training tools based on results from an international survey of CMR programs.

**Results:**

At present, 17 CMR programs have established a legal agreement to participate in GCMR, amongst them 10 have contributed CMR data, totaling 62,456 studies. There is currently a predominance of CMR centers with more than 10 years of experience (65%), and the majority are located in the United States (63%). The most common clinical indications for CMR have included assessment of cardiomyopathy (21%), myocardial viability (16%), stress CMR perfusion for chest pain syndromes (16%), and evaluation of etiology of arrhythmias or planning of electrophysiological studies (15%) with assessment of cardiomyopathy representing the most rapidly growing indication in the past decade. Most CMR studies involved the use of gadolinium-based contrast media (95%).

**Conclusions:**

We present the goals, mission and vision, infrastructure, preliminary results, and challenges of the GCMR.

**Trial registration:**

Identification number on ClinicalTrials.gov: NCT02806193. Registered 17 June 2016.

**Electronic supplementary material:**

The online version of this article (doi:10.1186/s12968-016-0321-7) contains supplementary material, which is available to authorized users.

## Background

Over the past decade, cardiovascular magnetic resonance (CMR) has become a key clinical imaging method for the evaluation of a wide range of heart and vascular diseases. A registry that fosters multicenter participation will gather evidence of the real-world diagnostic and therapeutic impact of CMR on patient care, key issues guiding future technical development, clinical adaptation, regulatory approval, and financial reimbursement. The European CMR Registry (EuroCMR) with now over 37,000 patients from 57 European centers has demonstrated CMR’s impact on clinical diagnosis and management in Europe [1, 2]. Given the worldwide clinical adaptation of CMR in the past decade, the Society for Cardiovascular Magnetic Resonance (SCMR) in 2013 initiated and has since continued to support the development of a global registry. The Global CMR Registry (GCMR) aims to promote evidence-based adoption of CMR into patient management by facilitating standardized data collection across many centers of diverse patient demographics and clinical outcomes, qualitative and quantitative CMR results, determination of the downstream impact of CMR on diagnostic and therapeutic thinking, and its cost-effectiveness.

## Methods/design

### Mission and vision of GCMR

The overarching vision of GCMR is to provide a central, representative collective platform to demonstrate the impact of clinical CMR applications on patient care and how CMR’s diagnostic and prognostic value impact patient management. Participation in GCMR is open to all CMR programs worldwide. Programs are encouraged to participate irrespective of their countries or regions, practice setting (e.g. academic, community), stage of CMR program development, or pre-existing CMR volume. It is the opinion of the GCMR steering committee that “real-world” data from clinical CMR practices will foster accurate cost-utility and cost-effectiveness analyses, with the vision of quantifying CMR’s impact over time towards improving the life expectancy and quality of life for patients with cardiovascular diseases. To succeed, GCMR has been set up as a platform that is inclusive and adaptable to promote collaboration amongst as many programs as possible, with data collection procedures that minimize the additional work burden of participating sites.

### GCMR organization

GCMR development is closely supported and supervised by the SCMR leadership. The organization of the GCMR includes a) the Chief Executive Officer and the Executive Committee of the SCMR, including its President, Vice President, Secretary-Treasurer, Vice Secretary-Treasurer, and Immediate Past-President, b) the GCMR committee, and c) a data management team. SCMR has not only provided the seed funding and support for the development of the infrastructure of GCMR website, but is directly coordinating the legal and contractual correspondence with all participating sites. Detailed and up-to-date information on GCMR, including its goals, vision and mission, leadership, roadmap of future GCMR development, data policy standards, template contractual agreements, current participating sites and investigators, lists of variables, and a sample Internal Review Board (IRB) protocol, can be found at the website http://gcmr-scmr.org.

The GCMR steering committee serves directly under the auspices of SCMR over a renewable 3-year term, overseeing all aspects of the development of this global registry. Additional file 1: Table S1 lists the current members of the GCMR steering committee [see Additional file 1: Table S1]. It consists of an international panel appointed by the SCMR from diverse disciplines and geographic regions. The steering committee serves to guide the development of registry policies and infrastructure, and utilization of the registry data. In addition, the steering committee makes executive decisions regarding research proposals and projects based on the scientific merits of the proposals. The data management team consists of clinician scientists, experts in information technology and webpage development, and a project manager. These members are responsible for advancing the GCMR database infrastructure, enrollment and training of participating sites, and harmonization of de-identified data.

### Roadmap of GCMR development

**Fig. 1:**
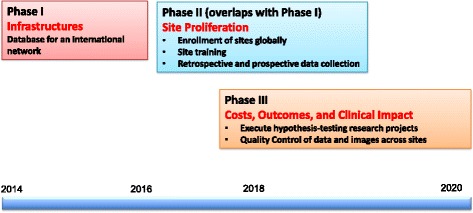
Phases of GCMR development in establishing database infrastructures, site recruitment, and assessment of clinical impact

The GCMR project plan calls for three distinct but overlapping phases. Figure 1 illustrates the projected development of GCMR over these phases. During the first phase, a web-based secure database infrastructure for creating an international network was developed and expanded. In the second phase, the current stage of development, the GCMR team focuses on the enrollment and training of CMR programs from diverse geographic regions. During this phase, the GCMR team first assesses the data collection methods and technical challenges of any given site, and then proposes a data contribution plan specific for the site. A data use policy document, a legal agreement between SCMR and the participating site, and an IRB protocol template have been prepared by a GCMR core team and are sent to each participating site to ensure a clear understanding of the goals and obligations of both parties. The third phase will commence when research concepts designed to utilize GCMR data are submitted to the steering committee for evaluation and approval. If the results of a research project are reported in a journal, sites that contributed significant amount of data to the project will be granted authorships. Although GCMR is currently in the second phase, aspects of the first phase (e.g. recruitment, training, website development) are ongoing and iterative.

### Phase I: infrastructure development

#### Key data variables and the GCMR website

A vital element in multicenter collaboration in GCMR lies in standardizing a list of key data variables that can be collected during clinical workflow of CMR imaging, balancing between data comprehensiveness and onerousness. A registry website of GCMR [www.gcmr-scmr.org] has been designed and implemented. The list of the current key GCMR variables (required and recommended) at the time of this writing can be found in the [see Additional file 2: Table S2] and at https://gcmr.bwh.harvard.edu/data/wiki_pages/10/database_variables.htm. The GCMR website also provides documents including the latest list of participating GCMR programs and case volume status, downloadable site-participation legal agreements, GCMR data policies, IRB protocol templates, and the GCMR vision and mission statements. The GCMR website supports uploading of formatted de-identified data fields spanning patient demographics, cardiac histories, CMR metrics including cardiac function, tissue characterization, pulse sequence descriptions and imaging protocols. In addition, it allows uploading and viewing of DICOM image files. The GCMR website performs automated anonymization of all protected health information in the DICOM headers during the uploading process. The GCMR registry has been registered on ClinicalTrials.gov (ID: NCT02806193).

#### GCMR Web based tools to facilitate site participation

From the inception of GCMR development, it was considered crucial to incorporate the process of data collection into the clinical workflow to reduce or minimize the time burden placed on the participating sites. In addition, the future success of the GCMR depends on an effective method of merging standardized data across all participating sites. To fulfill both requirements, the GCMR endorsed a non-profit web database (CMR Cooperative, https://cmrcoop.partners.org/) for collecting standardized PHI-free data at participating sites. Multi-level access privileges are administered at the individual sites’ level, which allows sites to use this web tool for local reporting and research purposes. Key features of CMR Cooperative include rapid collection of the most critical data variables for accurate and effective clinical reporting and contribution to GCMR. However, CMR Cooperative provides a host of other functions that a site can utilize in performing its own clinical or research activities, as follows:

- Data collection in patient demographics and study protocols relevant in major common CMR and CCT indications
- Data collection in cardiac events and diagnostic or therapeutic impact of imaging
- Data collection of concurrent cardiac imaging tests including storage of ECG records
- Data collection of segmental maps for perfusion, wall motion, late gadolinium enhancement, T1 and T2 mapping imaging
- Report generation for both CMR and cardiac computed tomography (CCT) studies
- An option of rapid report generation by a single-page data entry
- Site-specific administration of users’ privileges and account access
- Site-specific encryption key of all PHI, generated independently by each site
- Site-specific customization of reporting formats and other functions
- Site-specific unlimited downloading of its own latest datasets
- A scheduling module for tracking of CMR or CCT studies and corresponding study staff
- Criteria-based constructible search of a site’s own data
- Batch uploading of selected data variables
- Training manuals, videos, and recorded webinars

The Supplemental section [See Additional file 3: Figure S1, Additional file 4: Figure S2, Additional file 5: Figure S3, Additional file 6: Figure S4, Additional file 7: Figure S5, Additional file 8: Figure S6, Additional file 9: Figure S7, Additional file 10: Figure S8] illustrates a series of the selected webpages of CMR Cooperative. Participating sites can provide efficient clinical reporting as well as concurrently fulfilling the GCMR data requirement.

Other than using the GCMR-affiliated web database, sites may also contribute to GCMR by providing data that conform to the data formats specified by GCMR, per the key data variable list as illustrated [See Additional file 4: Figure S2] or in weblink https://gcmr.bwh.harvard.edu/data/wiki_pages/10/database_variables.htm. GCMR data management team also has harmonized data submitted by selected sites into the GCMR database and will continue to do so.

#### Data security

All data contributed by sites are collected in accordance with HIPAA or other privacy legislation in place in the countries of the respective contributing sites. Data collected and stored in GCMR does not contain any protected health information (PHI). A full description of PHI and guidance regarding methods for de-identification of PHI in accordance with the Privacy Rule of the Health Insurance Portability and Accountability Act (HIPAA) of 1996 can be found at http://www.hhs.gov/hipaa/for-professionals/privacy/special-topics/de-identification/. CMR Cooperative encrypts all PHI in transit (all access is provided exclusively via a secure HTTPS connection) and during data storage. In addition, a client-side encryption method [3] has been made available to sites to create and manage their own private encryption keys. For sites that utilize their own institution-developed software in data collection, all PHI were removed before data were submitted to GCMR for processing and storage in the repository. All GCMR data servers utilize open-source technologies: Ubuntu long term support (LTS) distribution of Linux, MySQL database, and Nginx web server. The web applications are built using the Ruby on Rails (RoR) application framework. The network connection is secured (128-bit encryption, TSL 1.2) and authenticated using AES_128_GCM and DHE_RSA as the key exchange mechanisms.

### Phase II: site proliferation

#### Institutional review and site initiation

While IRB approval may not be required for retrospective collection of de-identified data, the GCMR strongly recommends local IRB approval and all participating sites are expected to consult their local IRB prior to submitting any data. GCMR provides prospective sites with initiation packages including a password-secure account to its web database and associated training materials (instructional manual and video files), IRB application samples, and a list of key GCMR variables including their variable formats. In addition, the central GCMR team offers a series of webinars for site training purposes.

#### Legal agreement between each participating site and the SCMR

Each participating site is required to have the official agreement issued by the SCMR signed by its institutional signatory or designated representative. Approval of the terms of the agreement by a legal signatory at the site is considered a prerequisite for GCMR participation.

#### Policy towards authorships of manuscripts and funding support

##### Data use policy

A data use policy document has been distributed to all participating sites and researchers who are interested in applying for access to de-identified datasets. It is also available at https://gcmr.bwh.harvard.edu/about/research_goals. This policy document was prepared by the GCMR steering committee and approved by the executive committee of the SCMR. It serves as a governing guideline for GCMR’s policies in the areas of publication and participating sites’ rights and responsibilities to accessing GCMR’s database, and also describes the decision process used for the approval of sub-studies. Since GCMR is established under the auspices of the SCMR, any aspect of the data use policy of the GCMR must conform to the existing SCMR policies and bylaws. All researchers, regardless of whether his/her site has contributed data to GCMR, are encouraged to propose research projects that make use of de-identified GCMR data. Such requests for either sub-studies aiming at publications or grant proposals are to be made in writing in the form of a 1–2 page “request for sub-study” proposal, which will be evaluated by the GCMR steering committee. This evaluation will be based primarily on the level of scientific merit and the expected clinical impact of the study aims of the proposal. If approved, data access will be granted to the researchers for a specific mutually-agreed period determined primarily on the magnitude of the work involved, and only for the purposes of the grant proposal or sub-study. At the time of the sub-study submission, a publication plan is also required, detailing hypotheses, authorship, intellectual property created and timelines. Recommendations from the International Committee of Medical Journal Editors’ (ICMJE) regarding authorship and non-authorship will be followed to the fullest extent possible. If journals allow, the GCMR participants should be listed following the authors using the phrase “on behalf of the GCMR contributors” (www.icmje.org).

### Phase III: clinical impact, outcomes and costs

#### Research and quality control

The ultimate goals of GCMR are to determine the real-world value of CMR in disease diagnosis and management, and the impact of CMR on healthcare costs. Phase III will consist of the development and implementation of prospective projects designed to fulfill these goals. Participating programs will have the opportunity to submit research proposals to the steering committee for consideration. The steering committee will review the proposals and recommend those that meet criteria for scientific merit, clinical impact, novelty, and relevance to the goals of GCMR. Sites that contribute an adequate volume of data used for the research and data analysis will be considered for co-authorship in affiliated publications. Potential prospective projects include: assessment of the clinical impact of stress CMR perfusion imaging in patients with chest pain syndromes, an evaluation of the pertinence of the current appropriate use criteria (AUC); a comparison of the diagnostic and therapeutic impact of CMR against echocardiography and routine angiography in patients presenting with heart failure and unclear etiology; and the safety of gadolinium-based contrast media. Guidelines for quality control will also be conducted continuously and will allow for the more experienced sites to help guide the development of newer programs.

## Results

### Progress of GCMR to-date

**Fig. 2:**
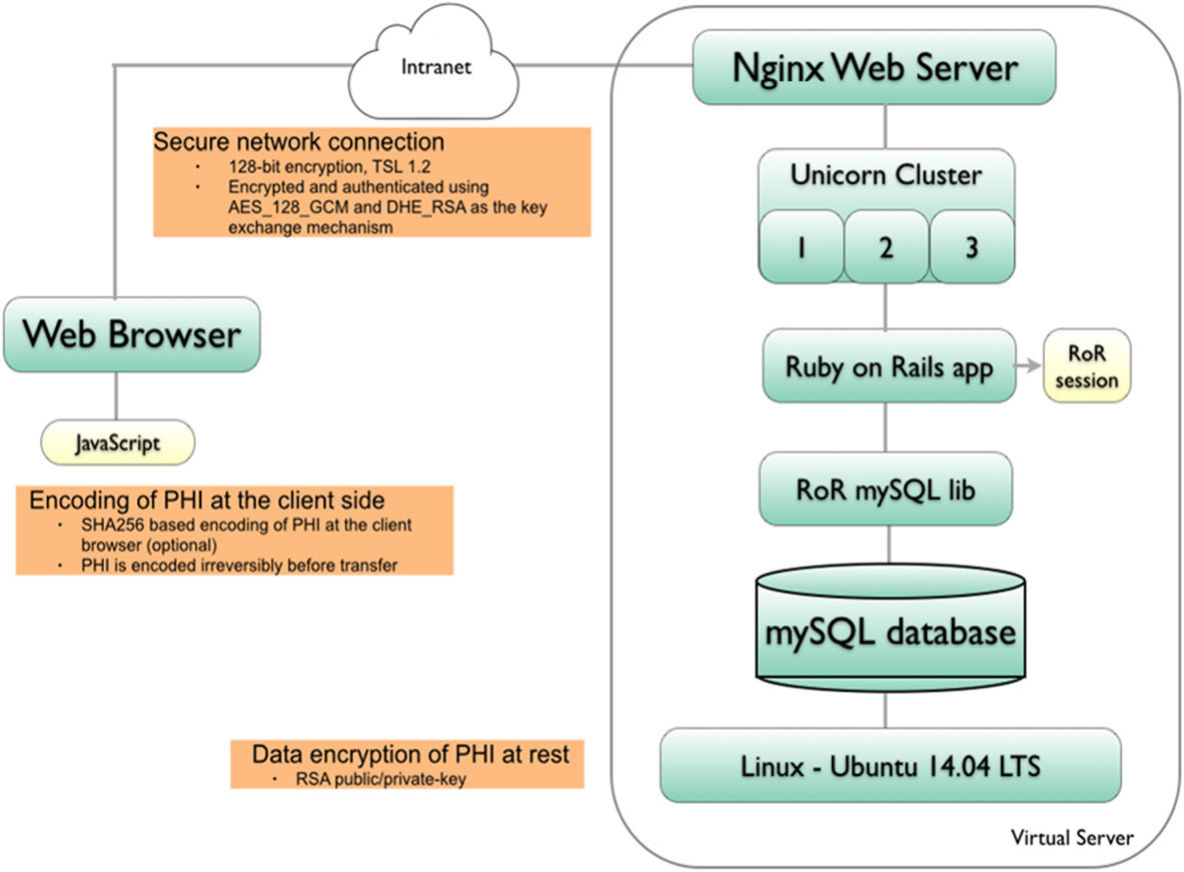
Security structures of GCMR web database

To date, phase I has been completed through the creation of a web-based database for an international network. Features of this database include a) CMR and cardiac CT capability, b) client-side encryption of all PHI using a site-defined security key, c) independent administration of multi-level user access and password control by each contributing site, d) rapid single-page data entry of all required key variable fields, e) report-generating capability to facilitate clinical workflow, and f) independent unrestricted access by each site to its own data. Site proliferation (phase II) and data collection of clinical impact, outcomes and costs (phase III) are currently underway. At the writing of this document, 45 centers were engaged in the enrollment process, amongst them 17 had signed the participating agreement. The countries of origin of these 17 programs include United States (*N* = 11), United Kingdom (*N* = 1), China (*N* = 1), Brazil (*N* = 1), South Africa (*N* = 1), India (*N* = 1), and New Zealand (*N* = 1). Eleven of the 17 sites (65%) were considered highly experienced with more than 10 years of clinical CMR performance. At present, 10 sites (9 of them highly experienced) have contributed clinical data: a total of 62,456 de-identified CMR studies from variable time periods between 2000 and 2015, have been collected and merged into the GCMR data registry. Table 1 shows the data contributions from the 10 sites. These contributing sites include programs from the United States of America (*n* = 8), the United Kingdom (*n* = 1), and China (*n* = 1) (Fig. 2).

**Table 1:** Current CMR volume in GCMR

CMR Program	Database Used	Years of CMR Contributed	Number of CMR Cases Contributed	Conformed to GCMR data format since year
Brigham and Women’s Hospital	GCMR-endorsed	2001–2015	10,537	2001
Central Utah Clinic	Institution developed	2002–2012	9,237	2016
National Institutes of Health	GCMR-endorsed	2001–2016	7,324	2015
Ohio State University	Institution developed	2004–2011	11,267	2016
Oklahoma Heart Institute	Institution developed	1999–2013	7,316	2016
University of Oxford	Institution developed	2002–2015	8,714	2016
St. Francis Hospital	Institution developed	2012–2015	2,141	2015
University of South Florida	GCMR-endorsed	2009–2015	1,886	2009
West China Hospital	GCMR-endorsed	2011–2015	3,060	2011
Wilford Hall Medical Center	GCMR-endorsed	2007–2015	974	2007
Total			62,456

### Demographic data of the current GCMR cohort

Table 2 demonstrates the demographic pattern of the cohort. The median age of the cohort was 55 years [range from 15 to 91] with a preponderance of males (68%). While the median left ventricular volumes (LVEDV and LVESV) and ejection fraction (LVEF) were within normal limits, CMR had been performed in patients with extreme values of LVEDV (50 ml - 560 ml), LVESV (30 ml – 350 ml), and LVEF (8 –91%).

**Table 2:** Demographic data of the current GCMR cohort

Characteristics of cases in GCMR			
Patient characteristics		Percentage of missing data	
		Whole cohort, *N* = 62,456	GCMR endorsed database, *N* = 23,781
Age (years), median (Q1, Q3)	55 (40, 68)	3%	0.001%
Female sex, %	31.6	3%	0.0004%
Height (m), median (Q1, Q3)	1.7 (1.6, 1.8)	14%	2%
Weight (kg), median (Q1, Q3)	79.5 (66.0, 93.8)	14%	2%
BSA (m^2^), median (Q1, Q3)	1.9 (1.6, 2.1)	14%	2%
Cardiac Function, median (Q1, Q3)			
LVEDV (ml)	145.0 (115.0, 182.0)	15%	10%
LVESV (ml)	57.7 (41.0, 83.0)	15%	10%
LVEF, calculated or estimated (%)	59.6 (51.2, 66.0)	10%	10%
LV Mass (gram)	118.0 (91.0, 154.0)	34%	19%
RVEDV (ml)	131.0 (96.0, 168.0)	62%	17%
RVESV (ml)	54.6 (35.0, 77.1)	62%	17%
LVEDVI (ml/m^2^)	79.6 (63.2, 110.0)	17%	11%
LVESVI (ml/m^2^)	33.2 (37.0, 23.5)	17%	11%
Cardiac History, %			
History of MI	13.6	15%	6%
History of PCI	12.7	16%	6%
History of CABG	5.5	16%	6%
History of HTN	41.8	15%	6%
History of DM	15.0	15%	6%
Rest wall motion abnormality, %	26.4	19%	9%
Abnormal late gadolinium enhancement, %	12.5	37%	9%

### Most common clinical indications and pattern of growth of CMR

**Fig. 3:**
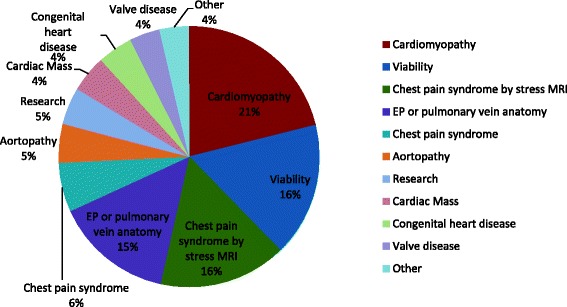
Most common CMR indications by ‘Appropriate Use Criteria’ categories

**Fig. 4:**
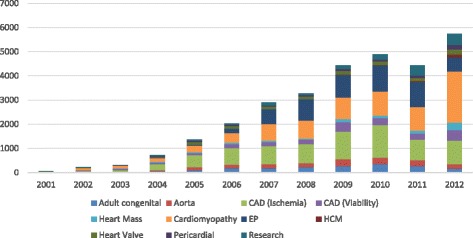
Growth of CMR indications in the GCMR cohort, 2001–2012. Average number of CMR studies across indications by year

Figure 3 illustrates the most common indications for CMR, using the indications listed in the recent appropriate use criteria guideline [4]. The indication for CMR was provided in 44,486 studies (71%) from 9 of the 10 current contributing sites. Assessment of cardiomyopathy, myocardial viability, planning of pulmonary venous isolation or electrophysiological ablation procedures, and assessment of chest pain syndromes using stress perfusion imaging represent the most common indications for CMR studies currently in the registry. Figure 4 illustrates the average number of CMR studies per program by years, stratified by various CMR indications. From the study data submitted to GCMR, there has been a progressive growth of clinical volume in the past decade, with key indications that demonstrated most growth include planning of electrophysiological ablation procedures and assessment of cardiomyopathy, which had almost tripled and quadrupled from 2006 to 2012, respectively.

### Gadolinium-based contrast media

**Fig. 5:**
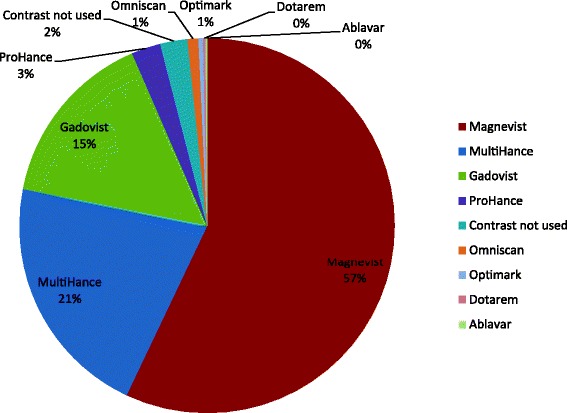
Distribution of the brands of contrast media used in GCMR CMR studies

Figure 5 illustrates the use of the various GBCA. GBCA use and dosing details were available from 53,742 CMR studies (86%) from 9 of the 10 sites. From this data, it was observed that the vast majority of studies (98%) within the GCMR cohort involved the use of a GBCA. Leading contrast agents used in the cohort studies included gadopentetate dimeglumine [Magnevist, Bayer AG, Leverkusen, Germany] (57%), gadobenate dimeglumine [Multihance, Bracco Imaging, Milan, Italy] (21%) and gadobutrol [Bayer AG, Leverkusen, Germany] (15%). All contributing programs followed a weight-based dosing algorithm, with 0.2 mmol/Kg as the most common cumulative dose used for each CMR study.

### CMR protocols and agreement with SCMR consensus guidelines

Table 3 illustrates the frequencies of the most commonly performed pulse sequences based on data collected by sites. Data regarding CMR pulse sequence was available from 46,426 CMR studies (74%) from 8 sites. Nearly all (95%) of CMR studies in the current cohort used cine steady-state free precession imaging. Late gadolinium enhancement imaging was the second most commonly utilized method, performed in 73% of studies. Pharmacological vasodilatation was used in 28%, with adenosine and regadenoson injections being the most often used. Interestingly, T1 mapping was reported performed in approximately 1 out of 4 studies. These patterns regarding the use of CMR pulse sequences are consistent with the recommendations of the SCMR [5, 6].

**Table 3:** Most common pulse sequence descriptions performed

Pulse sequence	Percentage (%)
Cine SSFP	92
T2W FSE	33
T2 Map	8
T1 Mapping	26
Double inversion Fast Spin Echo	16
Phase Contrast Imaging	18
T2 Star	10
Tagging	8
Coronary MRA using navigator	5
Late gadolinium enhancement	73
Rest Perfusion	64
Adenosine Vasodilating Perfusion	16
Dobutamine Stress Studies	2
Regadenoson Vasodilating Perfusion	8
Dipyridamole Vasodilating Perfusion	4

### Retrospective vs. Prospective data of the current GCMR cohort

As shown in Table 1, out of the 10 sites that had contributed data, 5 centers directly entered data using the GCMR endorsed database (CMR Cooperative) prospectively whereas the other 5 centers provided retrospective data collected using their own institutional database. The GCMR endorsed database (CMR Cooperative) has been established since September of 2008. The centers that use the GCMR endorsed database contributed 23,781 of the 62,456 (38%) CMR studies. All sites that are currently contributing data have agreed to collecting all key variables and to conform to the format of the variables defined by the GCMR. This condition is also a condition for all future sites to participate in GCMR. It appears that having 2 separate methods of contributing data to GCMR, by either directly using GCMR endorsed database or adherence to the data field formats, has encouraged more sites to participate by allowing sites to preserve their clinical workflow. It is anticipated that with the current efforts in standardizing data variables and variable formats, consistency of the pooled data will continue to increase.

### Data completeness

Completeness of data in the current GCMR pooled database collected from the 10 contributing sites is shown in Table 1. In the whole cohort, data fields that contained the highest percentage of reported missing data included right ventricular measurements (62%), left ventricular myocardial mass (34%), and presence or absence of late gadolinium enhancement (37%). Reasons for missing data in right ventricular measurements and left ventricular myocardial mass included technical problem in image acquisition, limited scanning per CMR indication, and routine protocoling per site. Reasons for omitted reporting of late gadolinium enhancement included the same reasons but in addition also the lack of a clinical indication for, or even the presence of a contraindication to gadolinium-based contrast agents (GBCA) administration. Across all data fields examined, percentages of missing data were lower amongst the 5 sites that used the GCMR endorsed database.

### Concerted data collection with CMR software vendors

Several CMR software vendors, including Medis Cardiovascular Imaging (Leiden, The Netherlands), Circle Cardiovascular Imaging (Calgary, Alberta), and Heart Imaging Technologies (Durham, North Carolina) have agreed to collaborate with GCMR by either aligning existing data fields or introducing key GCMR data variables into their software in future releases. It is anticipated this will allow additional sites to easily contribute data to GCMR via automated merging of datasets.

## Discussion

The current project represents the first registry with the goal of integrating data from CMR programs globally, independent of clinical practice setting and level of experience of the participating program. The GCMR has developed a HIPAA-compatible, nonprofit, web-based database structure to allow integrated data collection across CMR centers around the globe. Depending on the clinical or research needs of a given site, PHI is either removed or encrypted and stored in regional servers. Analytical and reporting components of our database are designed to meet the standards of current practice guidelines supported by the SCMR [5, 6]. This infrastructure allows data entry as a part of the clinical workflow with the goals of reducing the burden of redundant or onerous data entry. Given the complexity of CMR technology, multifaceted pulse sequence descriptions, and the wide range of clinical questions that CMR assesses, it is our opinion that this registry infrastructure will pave the path towards the growth of an integrated and a consistent body of evidence reflecting real-world data on CMR utilization and adoption. GCMR is the first of its kind to bring world-wide CMR practice patterns and clinical associations together in one unified database for purpose of evaluating the diagnostic impact and therapeutic guidance relevant to patient care.

While large-scale randomized prospective clinical trials will continue to provide the most robust evidence in guiding patient care, they can introduce selection bias, are costly, and in many clinical situations are impractical to conduct. Retrospective and prospective patient registries collecting real-world evidence can provide an alternative, complementary, and practical assessment for those conditions that are difficult to study in randomized trial settings. GCMR was designed to store a comprehensive range of data, which will enable researchers to evaluate various clinical outcome variables such as hospitalization, death, heart failure, arrhythmia and intervention as well as their relationship with commonly assessed clinical parameters, including cardiac function, chamber quantification, and myocardial perfusion. In contrast with clinical trials, which demand controlled conditions and tasks that are not usually reflective of daily clinical practice, patient registries provide a venue for collecting and storing data that correspond to parameters that are often routinely recorded. As such, patient registries are advantageous in that they are more amenable to mass-scale observational research and less prone to increasing the burden on participating CMR centers.

The EuroCMR Registry has prospectively enrolled over 37,000 patients from 57 centers in 15 European countries. Over the past decade, the EuroCMR Registry has led to new knowledge that is important to the clinical adoption of CMR: management changes were observed in approximately two-thirds of patients following a CMR scan [1]; the safety profile of pharmacological vasodilatation for stress CMR was demonstrated, as was the safety of GBCM at dosage appropriate for CMR [7]; and the prognostic implications of late gadolinium enhancement in hypertrophic cardiomyopathy patients [8] was investigated. More recently, a health economics study used the EuroCMR Registry data to demonstrate that a strategy of CMR as gatekeeper for invasive coronary angiograms can save costs when compared with a direct invasive strategy including fractional flow reserve measurements in patients with low to intermediate pre-test probability for obstructive coronary artery disease [9]. GCMR aims to learn from the successes of the EuroCMR Registry. While currently substantially less-developed than the EuroCMR Registry, GCMR is expected to continue to grow in CMR case volumes, geographic distribution and number of participating sites, and diversity of sites’ CMR experience.

### Challenges ahead

The GCMR faces several key challenges. First, retrospective data has a high proportion of missing entries as a portion of the data were collected prior to the establishment of a data variable list and variable formats. However, we observed substantially higher adherence rates of data entry amongst sites that had adopted the use of the GCMR endorsed database. Nonetheless, given that key variables such as major demographic factors, CMR indications, and contrast use are available in most of the sites, we believe that the current retrospective cohort represents a unique resource to assess CMR utilization patterns in the past and inform directions for growth in the future. Going forward, it is expected that participating sites will converge in their data collection methods prospectively, regardless of methods of data collection, and the quality of the data collected will improve. Second, the use of native languages in CMR reporting from various geographic regions poses an expected challenge as GCMR expands. An effort from an international panel of CMR experts is currently underway in translating the web based data structures into various languages.

## Conclusion

It is believed that the advantages of GCMR will be realized through its visions - becoming a universally representative CMR registry globally backed by the SCMR and a supportive and unifying data policy, user-friendly web data structures that are conducive to clinical workflow, and a goal of making a positive impact on patient care. GCMR provides the chance to acquire real-world, multi-dimensional evidence that can be used in many ways. Examples include a) to compare the clinical effectiveness of CMR with other imaging modalities, b) to conduct quality control of CMR images and data, which can be used to narrow the performance gap between nascent and experienced programs; c) to determine the cost-effectiveness of CMR when applied in important and common clinical scenarios; d) to study the impact on patient outcomes as compared to other imaging modalities. As an international registry, GCMR will offer opportunities to study variations across geographic regions, types of CMR center and CMR expertise in use of CMR protocols, performance, and clinical applications. Ultimately, the primary philosophy and goal of SCMR’s GCMR is to improve life expectancy and quality of life of patients with cardiovascular diseases.

## Additional files

Additional file 1: Table S1. List of current GCMR Steering Committee Members. (PDF 306 kb)
Additional file 2: Table S2. List of Key GCMR Variables Grouped by Data Category. (XLSX 28 kb)
Additional file 3: Figure S1. CMR Cooperative web database: User Access Management. Each participating site will assign its own site account administrator(s), who will administer the site’s users’ levels and durations of account access. (PDF 75 kb)
Additional file 4: Figure S2. CMR Cooperative web database: Patient Medical History. (PDF 175 kb)
Additional file 5: Figure S3. CMR Cooperative web database: Medications. A medication dictionary allows searching using either brand or generic drug names and automated entry of drug names into the database. In addition, medications are automatically categorized into specific drug classes for the ease of pooling multicenter data. (PDF 153 kb)
Additional file 6: Figure S4. CMR Cooperative web database: Quantitative CMR Data. Data entry for left and right ventricular measurements. “White” fields are data that need to be entered whereas “grey” fields are automatically calculated. (PDF 135 kb)
Additional file 7: Figure S5. CMR Cooperative web database: Clinical Outcomes. Common cardiovascular outcomes and their recurrent statuses can be collected. All dates are encrypted by a site’s own created encryption key. (PDF 99 kb)
Additional file 8: Figure S6. CMR Cooperative web database: Segmental Myocardial Perfusion by CMR. Collection of segmental perfusion defects according to the AHA 17-segmental model, during stress and rest hemodynamic states. (PDF 173 kb)
Additional file 9: Figure S7. CMR Cooperative web database: Segmental Myocardial Viability. Collection of segmental myocardial viability according to the AHA 17-segmental model. (PDF 110 kb)
Additional file 10: Figure S8. CMR Cooperative web database: A Sample Cardiac Computed Tomography (CCT) Page. (PDF 195 kb)

## Abbreviations

CMR: Cardiovascular magnetic resonance
GBCA: Gadolinium-based contrast agent
GCMR: Global cardiovascular magnetic resonance registry
HIPAA: Health insurance portability and accountability act
SCMR: Society for cardiovascular magnetic resonance
