# Supplementary material for: The global cardiovascular magnetic resonance registry (GCMR) of the society for cardiovascular magnetic resonance (SCMR): its goals, rationale, data infrastructure, and current developments
Source: J Cardiovasc Magn Reson. 2017 Jan 20;19:23. doi: 10.1186/s12968-016-0321-7 (PMC5303267; doi:10.1186/s12968-016-0321-7)
Supplement: Additional file 1: Table S1. — List of current GCMR Steering Committee Members. (PDF 306 kb) [file 12968_2016_321_MOESM1_ESM.pdf]

**Additional file 1: Table S1: GCMR Steering Committee Members:**

| GCMR Steering Committee Members | | | |
|---|---|---|---|
| **Name** | **Role** | **Occupation** | **Location** |
| Raymond Kwong | Chair | Cardiologist | United States |
| Erik Schelbert | Vice-Chair | Cardiologist | United States |
| Uma Valeti | Coordinator | Cardiologist | United States |
| Subha Raman | Member | Cardiologist | United States |
| Steffen Petersen | Member | Cardiologist | United Kingdom |
| Scott Flamm | Member | Radiologist | United States |
| Ted Martin | Member | Cardiologist | United States |
| Alistair Young | Member | Scientist | New Zealand |
| Ricardo Cury | Member | Radiologist | United States |
