# Supplementary material for: The global cardiovascular magnetic resonance registry (GCMR) of the society for cardiovascular magnetic resonance (SCMR): its goals, rationale, data infrastructure, and current developments
Source: J Cardiovasc Magn Reson. 2017 Jan 20;19:23. doi: 10.1186/s12968-016-0321-7 (PMC5303267; doi:10.1186/s12968-016-0321-7)

Additional file 3: Figure S1
CMR Cooperative web database: User Access Management

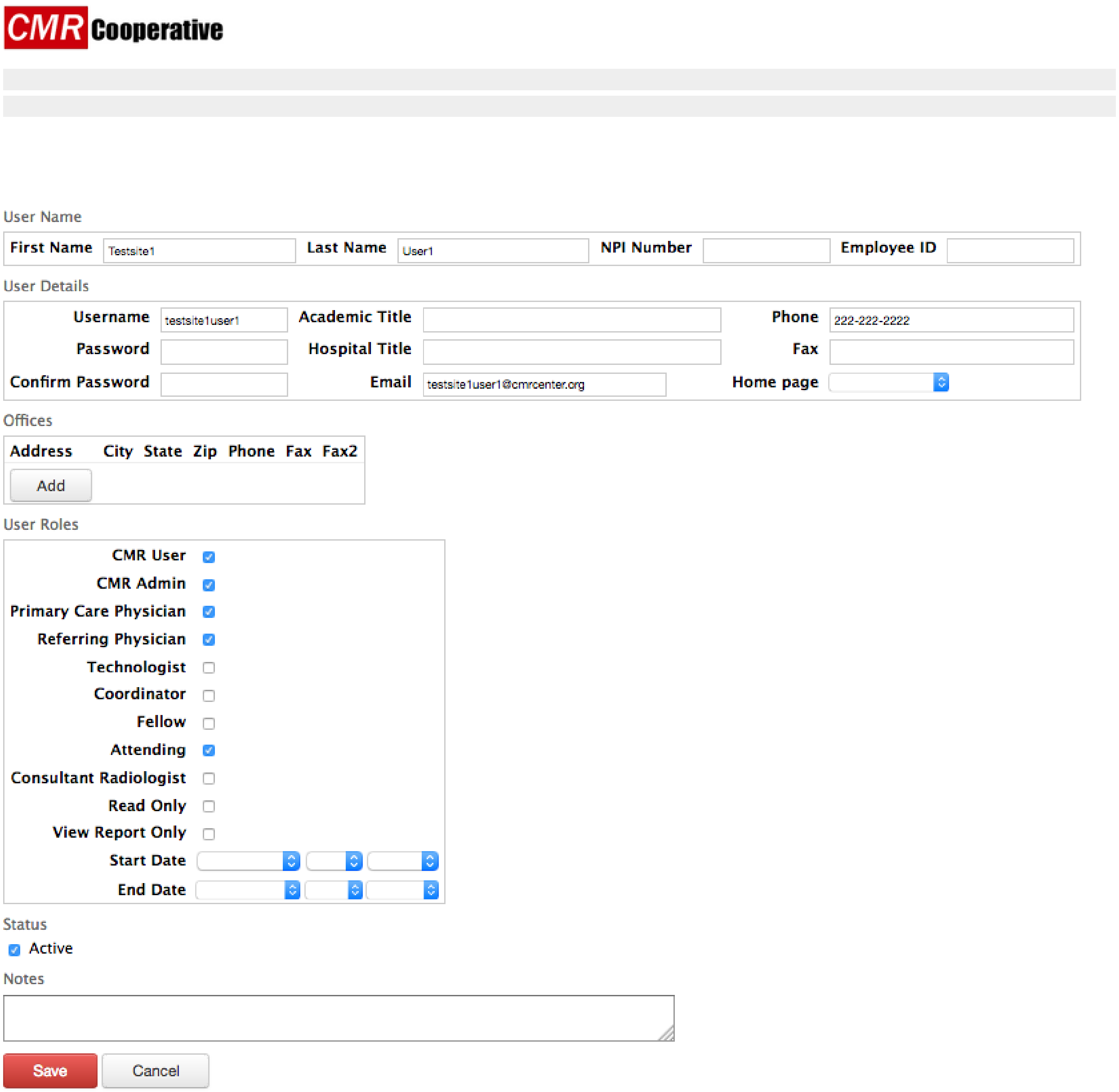

Supplement: Additional file 3: Figure S1. — CMR Cooperative web database: User Access Management. Each participating site will assign its own site account administrator(s), who will administer the site’s users’ levels and durations of account access. (PDF 75 kb) [file 12968_2016_321_MOESM3_ESM.pdf]
