# Supplementary material for: The global cardiovascular magnetic resonance registry (GCMR) of the society for cardiovascular magnetic resonance (SCMR): its goals, rationale, data infrastructure, and current developments
Source: J Cardiovasc Magn Reson. 2017 Jan 20;19:23. doi: 10.1186/s12968-016-0321-7 (PMC5303267; doi:10.1186/s12968-016-0321-7)

Additional file 5: Figure S3
CMR Cooperative web database: Medications

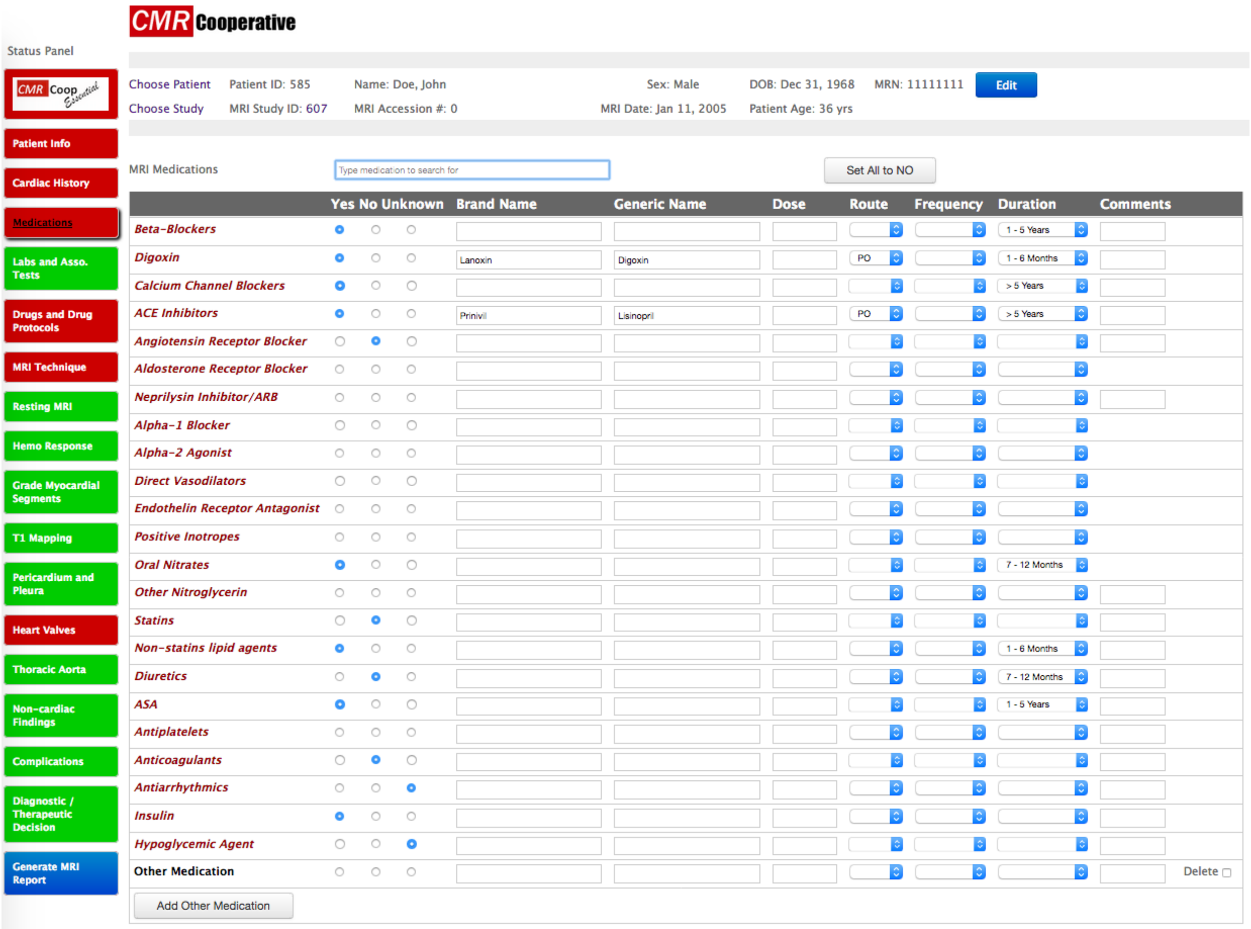

Supplement: Additional file 5: Figure S3. — CMR Cooperative web database: Medications. A medication dictionary allows searching using either brand or generic drug names and automated entry of drug names into the database. In addition, medications are automatically categorized into specific drug classes for the ease of pooling multicenter data. (PDF 153 kb) [file 12968_2016_321_MOESM5_ESM.pdf]
