# Supplementary material for: The global cardiovascular magnetic resonance registry (GCMR) of the society for cardiovascular magnetic resonance (SCMR): its goals, rationale, data infrastructure, and current developments
Source: J Cardiovasc Magn Reson. 2017 Jan 20;19:23. doi: 10.1186/s12968-016-0321-7 (PMC5303267; doi:10.1186/s12968-016-0321-7)

Additional file 6: Figure S4
CMR Cooperative web database: Quantitative CMR Data

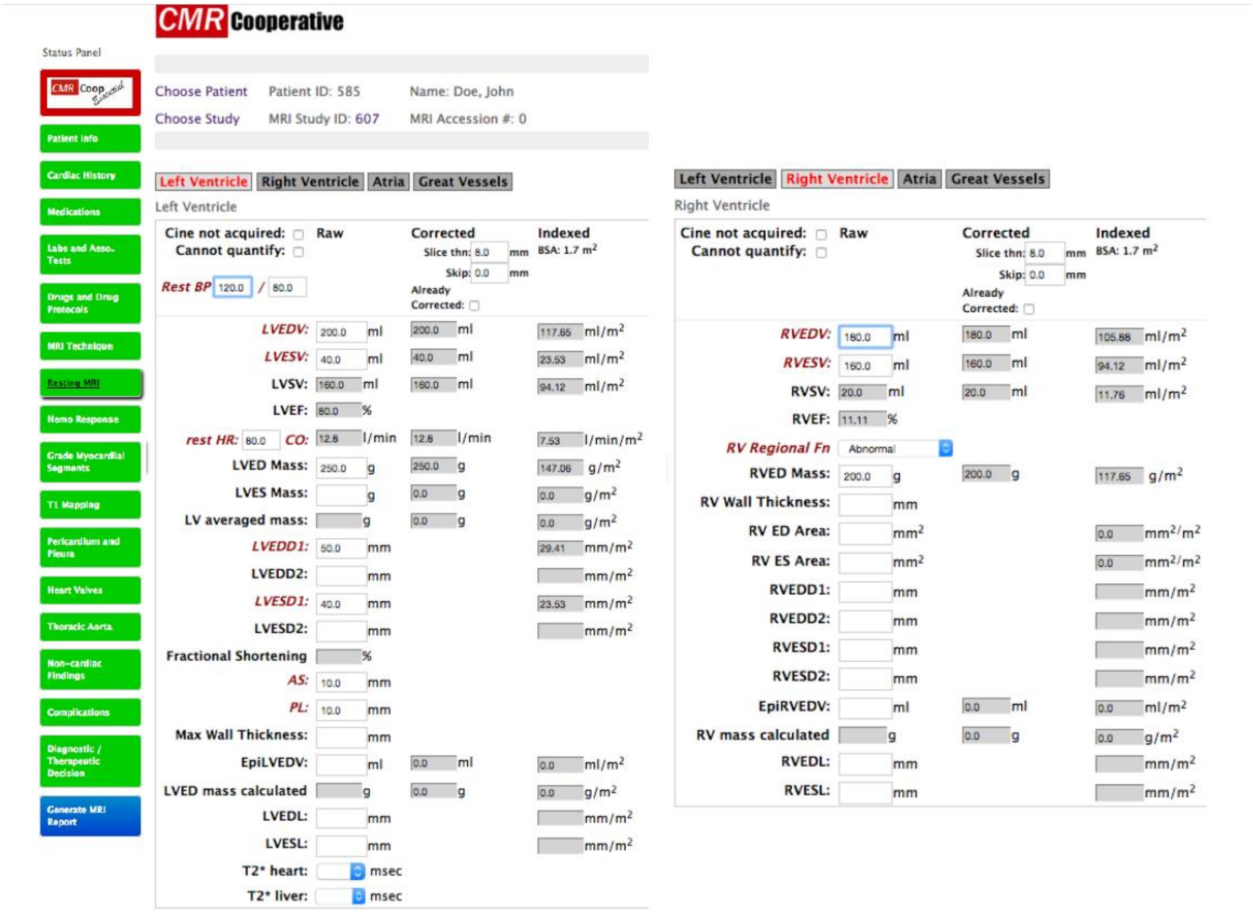

Supplement: Additional file 6: Figure S4. — CMR Cooperative web database: Quantitative CMR Data. Data entry for left and right ventricular measurements. “White” fields are data that need to be entered whereas “grey” fields are automatically calculated. (PDF 135 kb) [file 12968_2016_321_MOESM6_ESM.pdf]
