# Supplementary material for: The global cardiovascular magnetic resonance registry (GCMR) of the society for cardiovascular magnetic resonance (SCMR): its goals, rationale, data infrastructure, and current developments
Source: J Cardiovasc Magn Reson. 2017 Jan 20;19:23. doi: 10.1186/s12968-016-0321-7 (PMC5303267; doi:10.1186/s12968-016-0321-7)
Supplement: Additional file 7: Figure S5. — CMR Cooperative web database: Clinical Outcomes. Common cardiovascular outcomes and their recurrent statuses can be collected. All dates are encrypted by a site’s own created encryption key. (PDF 99 kb) [file 12968_2016_321_MOESM7_ESM.pdf]

Additional file 7: Figure S5
CMR Cooperative web database: Clinical Outcomes

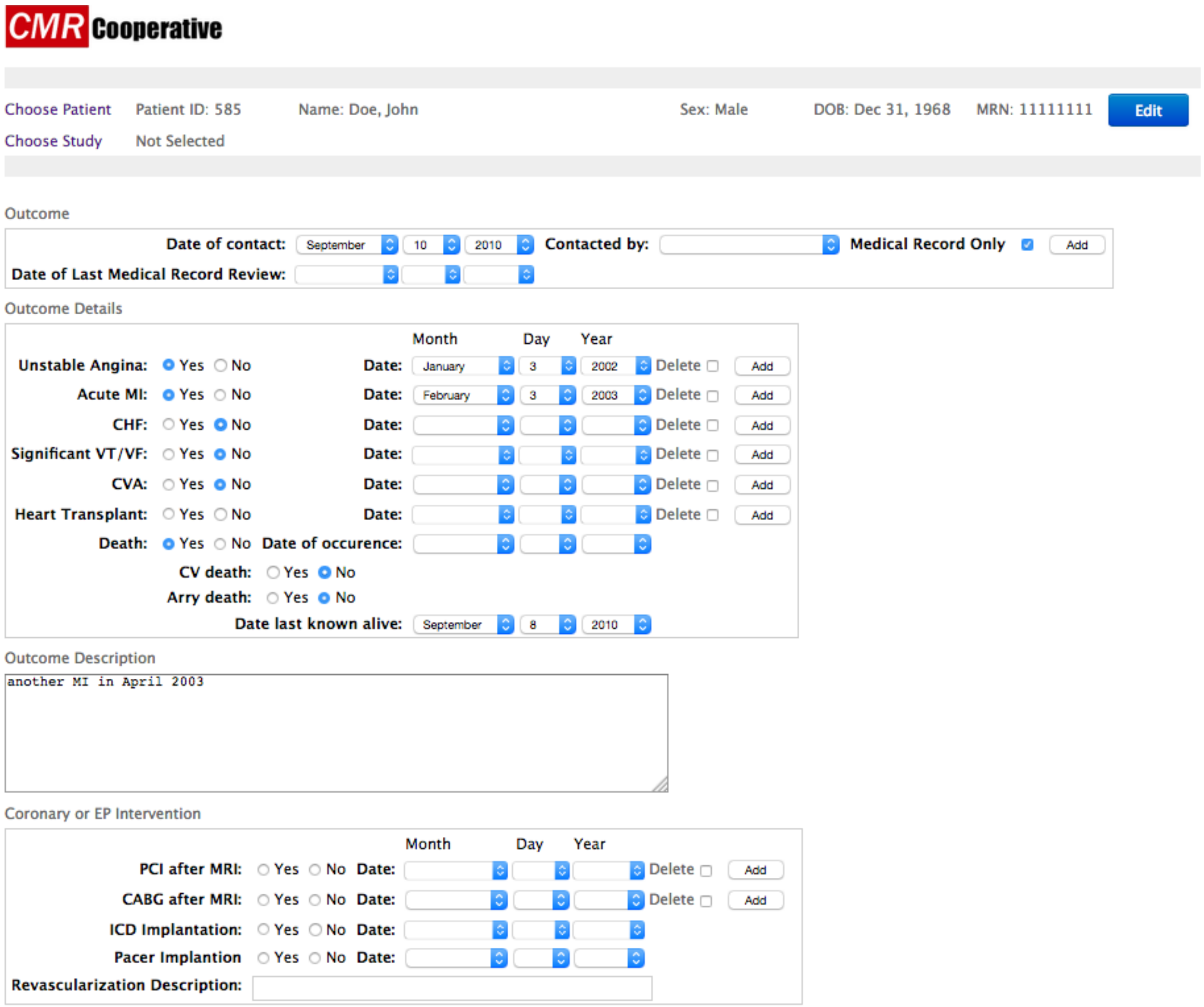

Choose Patient | Patient ID: 585 | Name: Doe, John | Sex: Male | DOB: Dec 31, 1968 | MRN: 11111111 | Edit

Choose Study | Not Selected

Outcome

Date of contact: September 10 2010 **Contacted by:** **Medical Record Only** ☑ Add

Date of Last Medical Record Review:

Outcome Details

		Date	Month	Day	Year		
**Unstable Angina:**	◉ Yes ○ No	**Date:**	January	3	2002	Delete ☐	Add
**Acute MI:**	◉ Yes ○ No	**Date:**	February	3	2003	Delete ☐	Add
**CHF:**	○ Yes ◉ No	**Date:**				Delete ☐	Add
**Significant VT/VF:**	○ Yes ◉ No	**Date:**				Delete ☐	Add
**CVA:**	○ Yes ◉ No	**Date:**				Delete ☐	Add
**Heart Transplant:**	○ Yes ○ No	**Date:**				Delete ☐	Add
**Death:**	◉ Yes ○ No	**Date of occurence:**					
**CV death:**	○ Yes ◉ No						
**Arry death:**	○ Yes ◉ No						
		**Date last known alive:**	September	8	2010		

Outcome Description

another MI in April 2003

Coronary or EP Intervention

		Date	Month	Day	Year		
**PCI after MRI:**	○ Yes ○ No	**Date:**				Delete ☐	Add
**CABG after MRI:**	○ Yes ○ No	**Date:**				Delete ☐	Add
**ICD Implantation:**	○ Yes ○ No	**Date:**					
**Pacer Implantion**	○ Yes ○ No	**Date:**					

Revascularization Description:
