# Supplementary figures and images for: The global cardiovascular magnetic resonance registry (GCMR) of the society for cardiovascular magnetic resonance (SCMR): its goals, rationale, data infrastructure, and current developments
Source: J Cardiovasc Magn Reson. 2017 Jan 20;19:23. doi: 10.1186/s12968-016-0321-7 (PMC5303267; doi:10.1186/s12968-016-0321-7)

Additional file 8: Figure S6

CMR Cooperative web database: Segmental Myocardial Perfusion by CMR

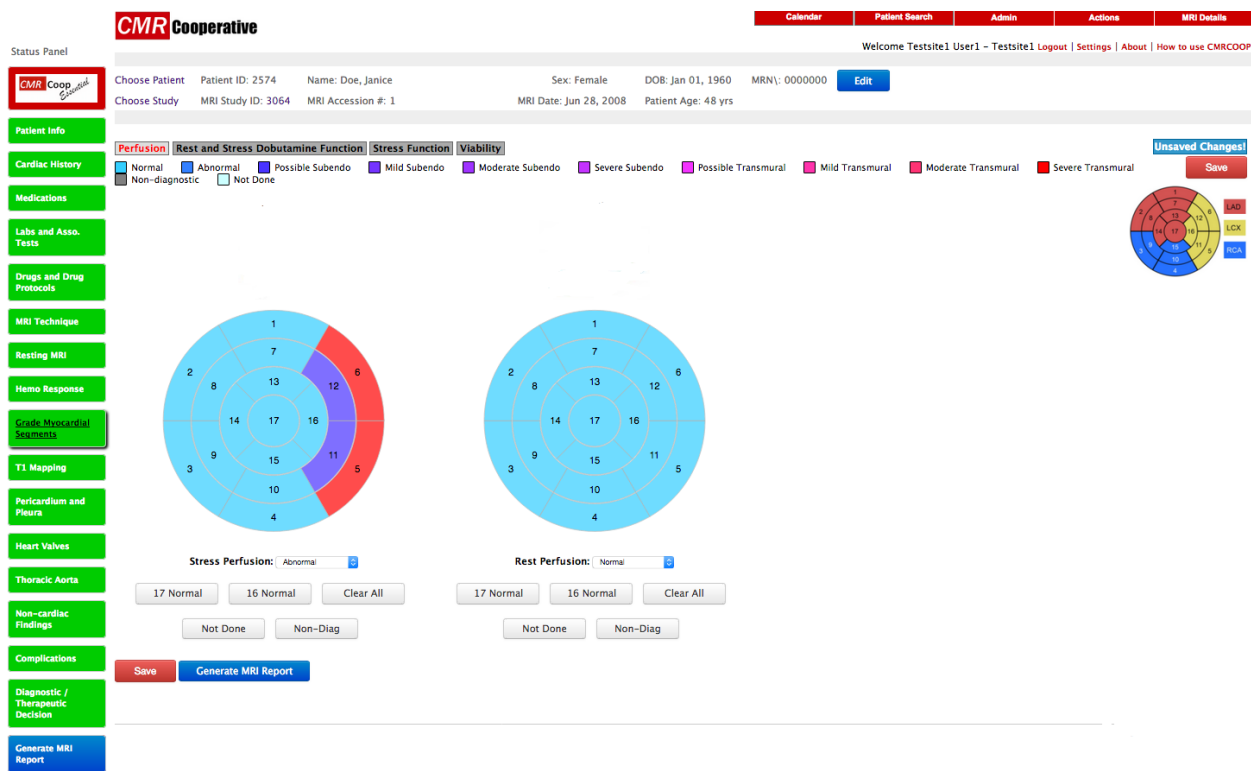

Supplement: Additional file 8: Figure S6. — CMR Cooperative web database: Segmental Myocardial Perfusion by CMR. Collection of segmental perfusion defects according to the AHA 17-segmental model, during stress and rest hemodynamic states. (PDF 173 kb) [file 12968_2016_321_MOESM8_ESM.pdf]
