# Supplementary material for: The global cardiovascular magnetic resonance registry (GCMR) of the society for cardiovascular magnetic resonance (SCMR): its goals, rationale, data infrastructure, and current developments
Source: J Cardiovasc Magn Reson. 2017 Jan 20;19:23. doi: 10.1186/s12968-016-0321-7 (PMC5303267; doi:10.1186/s12968-016-0321-7)

Additional file 9: Figure S7

CMR Cooperative web database: Segmental Myocardial Viability

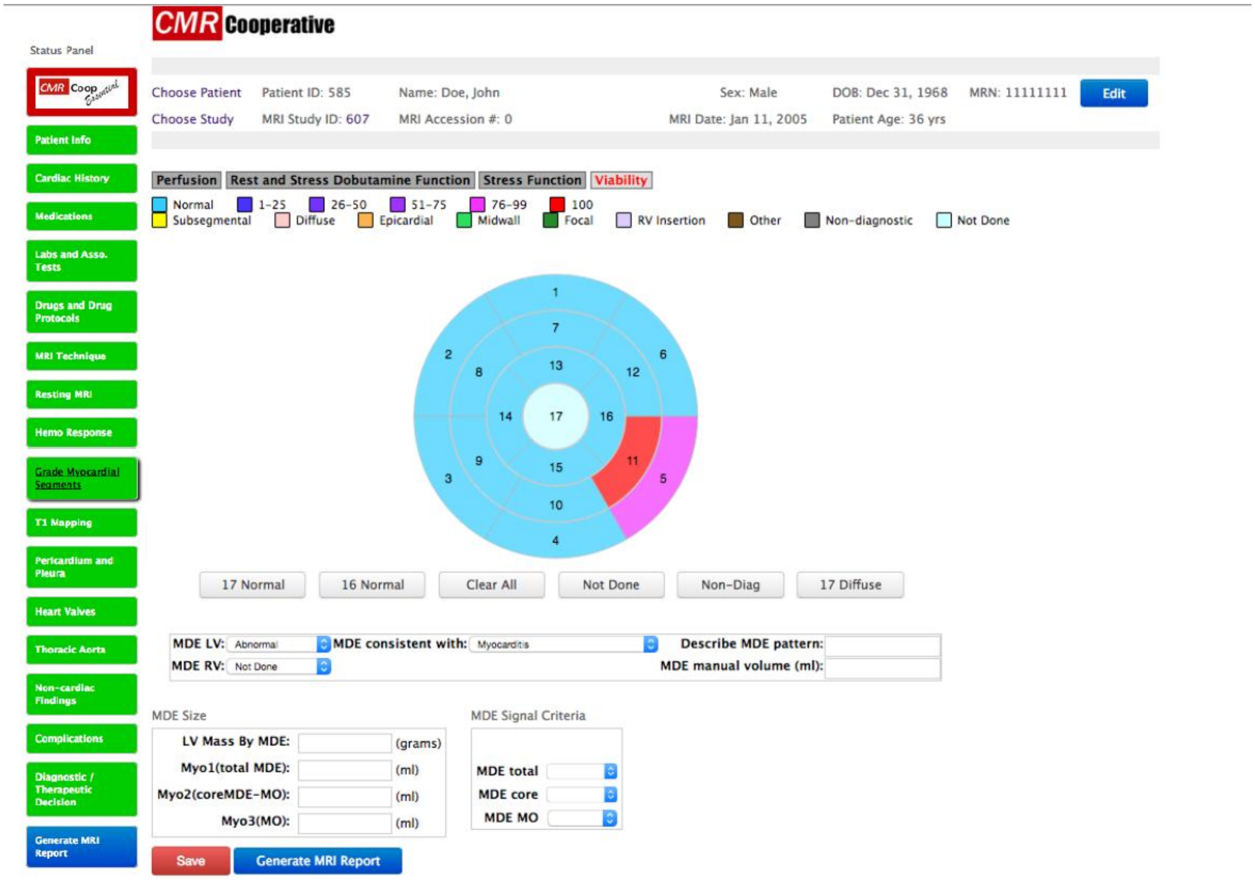

Supplement: Additional file 9: Figure S7. — CMR Cooperative web database: Segmental Myocardial Viability. Collection of segmental myocardial viability according to the AHA 17-segmental model. (PDF 110 kb) [file 12968_2016_321_MOESM9_ESM.pdf]
