# Supplementary material for: The global cardiovascular magnetic resonance registry (GCMR) of the society for cardiovascular magnetic resonance (SCMR): its goals, rationale, data infrastructure, and current developments
Source: J Cardiovasc Magn Reson. 2017 Jan 20;19:23. doi: 10.1186/s12968-016-0321-7 (PMC5303267; doi:10.1186/s12968-016-0321-7)

Additional file 10: Figure S8
CMR Cooperative web database: A Sample Cardiac Computed Tomography (CCT) Page

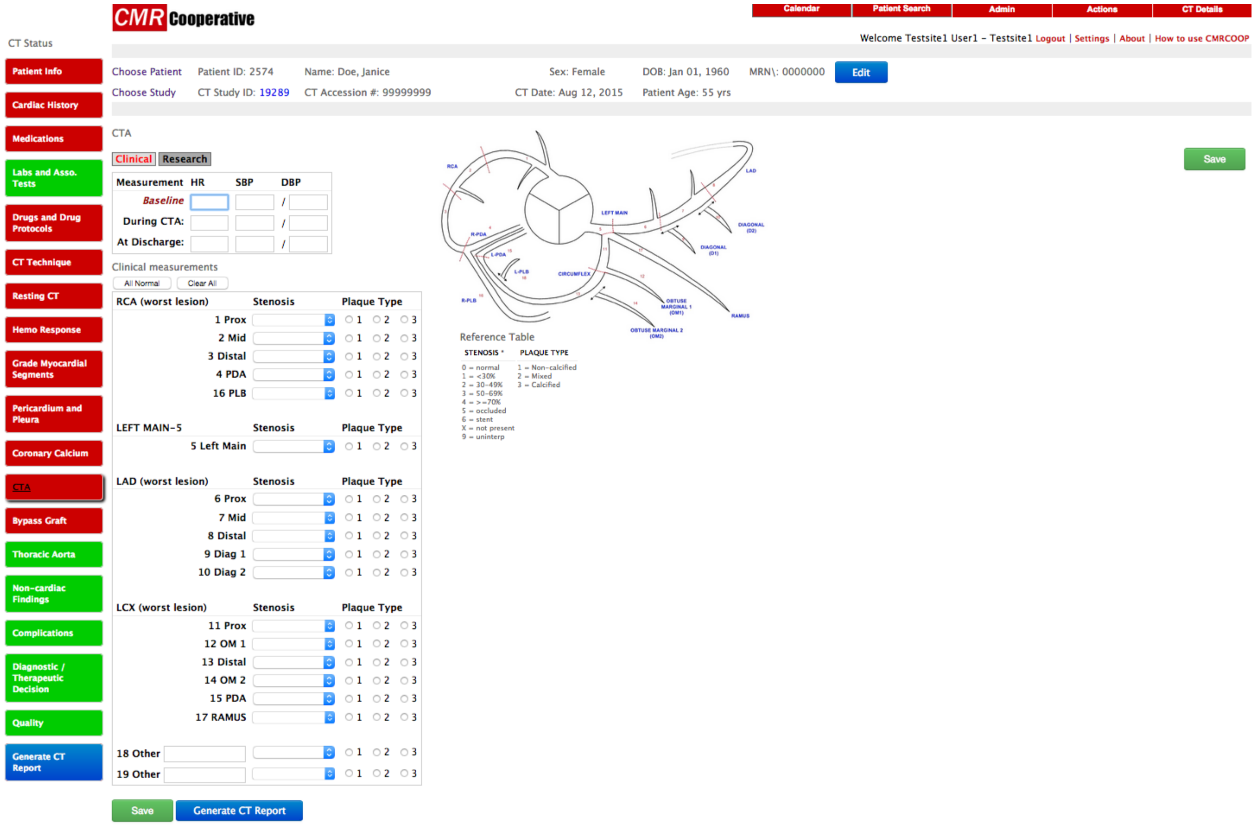

Supplement: Additional file 10: Figure S8. — CMR Cooperative web database: A Sample Cardiac Computed Tomography (CCT) Page. (PDF 195 kb) [file 12968_2016_321_MOESM10_ESM.pdf]
